# Origins and Function of VL30 lncRNA Packaging in Small Extracellular Vesicles: Implications for Cellular Physiology and Pathology

**DOI:** 10.3390/biomedicines9111742

**Published:** 2021-11-22

**Authors:** Stefania Mantziou, Georgios S. Markopoulos

**Affiliations:** 1 Haematology Laboratory-Unit of Molecular Biology, University Hospital of Ioannina, 45110 Ioannina, Greece; stefanimantz@gmail.com; 2 Neurosurgical Institute, Faculty of Medicine, University of Ioannina, 45110 Ioannina, Greece

**Keywords:** small extracellular vesicles, VL30 lncRNA, non-coding RNAs, cancer, SFPQ

## Abstract

Long non-coding RNAs (lncRNAs) have emerged during the post-genomic era as significant epigenetic regulators. Viral-like 30 elements (VL30s) are a family of mouse retrotransposons that are transcribed into functional lncRNAs. Recent data suggest that VL30 RNAs are efficiently packaged in small extracellular vesicles (SEVs) through an SEV enrichment sequence. We analysed VL30 elements for the presence of the distinct 26 nt SEV enrichment motif and found that SEV enrichment is an inherent hallmark of the VL30 family, contained in 36 full-length elements, with a widespread chromosomal distribution. Among them, 25 elements represent active, present-day integrations and contain an abundance of regulatory sequences. Phylogenetic analysis revealed a recent spread of SEV-VL30s from 4.4 million years ago till today. Importantly, 39 elements contain an SFPQ-binding motif, associated with the transcriptional induction of oncogenes. Most SEV-VL30s reside in transcriptionally active regions, as characterised by their distribution adjacent to candidate cis-regulatory elements (cCREs). Network analysis of SEV-VL30-associated genes suggests a distinct transcriptional footprint associated with embryonal abnormalities and neoplasia. Given the established role of VL30s in oncogenesis, we conclude that their potential to spread through SEVs represents a novel mechanism for non-coding RNA biology with numerous implications for cellular homeostasis and disease.

## 1. Introduction

Based on the latest Gencode reference annotation, the mouse genome contains >13,000 long non-coding RNAs (lncRNAs) [1]. Non-coding RNAs’ annotation and functional characterisation are critical to understand their contribution to physiological and pathological processes. Among the most acknowledged functions of lncRNAs is their role in epigenetic gene regulation through several mechanisms, such as of histone modifications, transcription factor recruitment, mRNA stability and miRNA occupancy [2]. Given the diverse biological roles of lncRNAs, they participate in several pathophysiological processes [3]. Among the most studied examples are those of ANRIL (Antisense Non-coding RNA in the INK4 Locus), HOTTIP (HOXA transcript at the distal tip) and XIST (X-inactive specific transcript) lncRNAs [4,5,6]. lncRNA ANRIL recruits the polycomb repressive complex in cell cycle regulatory genes, an action that is associated with cancer induction. HOTTIP, another oncogenic lncRNA, activates HOXA genes and is involved in leukemogenesis. XIST lncRNA has multiple roles in developmental X-chromosome inactivation and is also implicated in cancer induction. Recent studies have systematically verified the impactful physiological and pathological roles of lncRNAs as part of gene regulatory networks that also include microRNAs [7,8,9].

One of the known functional lncRNAs in the mouse genome is the RNA transcript from viral-like 30 elements (VL30s) that was characterised as early as 40 years ago [10]. VL30 RNA is a lncRNA with the capacity of packaging in type-C retroviral particles and is competent for reverse transcription and retrotransposition [11]. Our research team has previously calculated that VL30s appeared in the mouse germline 17.2 million years ago and have since spread through consecutive retrotransposition events [11]. Today, the reference mouse genome contains 372 VL30 sequences, categorised as 86 full-length and 49 truncated copies as well as 237 solo LTRs (long terminal repeats) with discrete chromosomal distribution [11]. VL30s contain in their LTRs a plethora of regulatory sequences, allowing their regulation by several stimuli, such as oncogenic and oxidative stress, classifying VL30s to be transcriptionally regulated as early response genes [12,13,14,15,16,17].

VL30 lncRNAs may affect cellular physiology by at least two distinct mechanisms. First, the vast majority of VL30 RNAs contain sequences that render them retrotransposition-competent [18]. VL30 retrotransposition is a highly mutagenic phenomenon that is associated with genetic plasticity and epigenetic deregulation that may lead to epithelial-to-mesenchymal transition and cancer stem cell formation [19]. An additional property of VL30 RNA is the direct binding to SFPQ (Splicing Factor Proline And Glutamine Rich), with a physiological role in steroidogenesis [20]. However, abnormal up-regulation of VL30 RNA may lead to carcinogenesis by SFPQ-dependent oncogene activation [21]. Based on recent results, we have proved that SFPQ binding is a universal feature of the VL30 family, with 83/86 of full-length elements containing at least one SFPQ-binding motif [11]. In summary, VL30 lncRNA is implicated in (patho)physiology with at least two mechanisms, competence for retrotransposition and SFPQ binding, acting as both genetic and epigenetic regulators in homeostasis and disease. 

Small extracellular vesicles (SEVs) are created by cells as a delivery mechanism of proteins, lipids or nucleic acids and as a medium of extracellular communication. Upon their discovery, SEVs were considered as a waste disposal mechanism. Today, SEVs have emerged as major players in several cellular processes, including immunoregulation, CNS development and homeostasis, tissue regeneration, inflammation and coagulation [22]. A recent report by Barrios et al. [23] quantified the RNA expression in SEVs derived from mouse dendritic cells. The most enriched RNAs (>200-fold abundance compared to cellular RNAs) were found to be VL30 lncRNAs. The authors have systematically analysed exosome-containing VL30 RNAs to find a distinct 26-nucleotide motif that is associated with SEV loading and an interferon type I response. Among the main conclusions from this seminal study was that the enrichment of VL30s RNAs into SEVs in conjunction with an immunostimulatory effect leads to their removal from the cell. This mechanism may act in general as a cellular “garbage-bin” to avoid potential toxic effects from non-coding RNAs and avoid autoinflammation. 

The aim of the current report was to find the origins and functions of VL30s that are packaged in SEVs and to establish whether they are a distinct group within the VL30 family. Towards this aim, we performed the analysis of phylogenetic and genomic distribution and enriched gene pathways and networks data. Our results support that the SEV enrichment motif occurred 4.4 million years ago (MYA) and spread through retrotransposition to at least 40 elements. Network analysis suggest a stress-associated transcriptional footprint. We conclude that enrichment into SEVs leads to the horizontal transfer of oncogenic VL30 lncRNAs with apparent implications in cellular pathophysiology.

## 2. Materials and Methods 

### 2.1. Sequence Analysis

Blast Like Alignment tool (BLAT) web interface in the UCSC Genome Browser (http://genome.ucsc.edu/ accessed on 14 September 2021) was used for finding SEV enrichment motifs in the latest assembly (mm39) of the mouse genome [24,25,26]. Genomic coordinates of SEV-VL30s were obtained using UCSC Table browser [27]. Basic Local Alignment Search tool (BLAST) [28] implementation in NCBI website (https://blast.ncbi.nlm.nih.gov/Blast.cgi accessed on 14 September 2021) was used to query for the presence of SEV enrichment motifs in the genomes of the *Mus* genus. The chromosomal distribution of SEV-VL30s was depicted in the Ensembl Genome Browser (https://ensembl.org/ accessed on 14 September 2021) [29,30]. The presence of candidate cis-regulatory elements by ENCODE [31] was performed in UCSC Table browser. Genomic Regions Enrichment of Annotations Tool (GREAT) [32] web implementation (https://great.stanford.edu/great/ accessed on 5 October 2021) was used to annotate SEV-VL30s in relation to transcription start sites (TSSs) of mouse genes. GREAT associates genomic regions with their nearby genes and applies the gene annotations to the regions, based on binomial and hypergeometric statistical tests, over regions and genes, respectively. Association is two-step. Every gene is assigned a regulatory domain, and then, each region is tested whether it overlaps with gene regulatory domains.

### 2.2. Molecular Phylogenetic Analysis

Multiple sequence alignment was conducted using the MUSCLE algorithm [33], as implemented in the European Bioinformatic Institute website (https://www.ebi.ac.uk/Tools/msa/muscle/ accessed on 5 October 2021). The phylogenetic tree was constructed following evolutionary history inference using the Neighbour-Joining method [34]. The evolutionary distances were computed using the Maximum Composite Likelihood method [35] and are in the units of the number of base substitutions per site. This analysis involved 40 nucleotide sequences. There were a total of 1130 positions in the final dataset. Evolutionary analyses were conducted in MEGA11 [36]. The phylogenetic tree of the *Mus* genus was generated using TimeTree [37] web interface (http://www.timetree.org/ accessed on 5 October 2021).

### 2.3. Pathway and Network Analysis

Network analysis was performed in Genemania, a binary classification algorithm for network construction [38,39], in the web server (https://genemania.org/ accessed on 6 October 2021), using Gene Ontology-based weighting methods. Enriched pathway analysis was performed in Enrichr [40,41] (https://maayanlab.cloud/Enrichr/ accessed on 6 October 2021), using default settings. Briefly, the gene set of interest was compared with preset gene set libraries in the Enrichr server, for significant correlations (*p* < 0.05) based on the Fisher exact test. The results were viewed using Appyters [42]. 

## 3. Results

### 3.1. SEV Enrichment Motif Is a Universal Feature of VL30 Family

**Figure 1:**
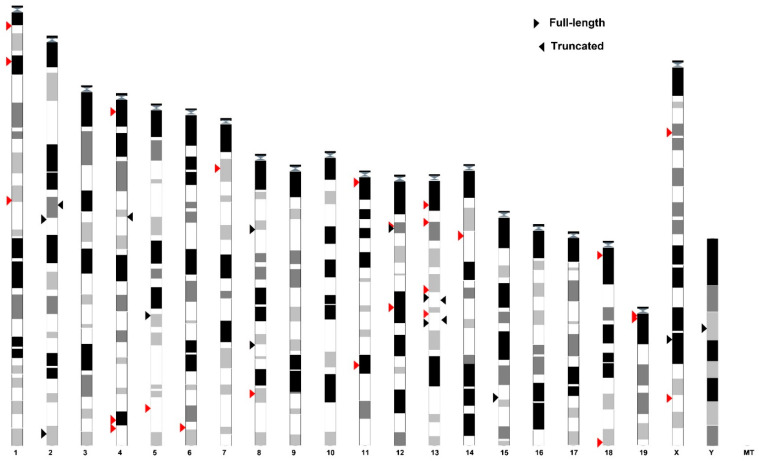
Genomic distribution and features of SEV-VL30s. Arrows on the left or right side of chromosomes indicate full-length or truncated VL30 elements, respectively. Active elements (contemporary integrations), as described in [11], are indicated by red arrows.

The efficient packaging of VL30 lncRNAs in small extracellular vesicles (SEVs) is associated with the presence of a 26-nt-long preserved SEV enrichment motif [23]. We analysed VL30 elements for the presence of a 26-nt SEV motif consensus sequence (AGATCGTGGGTTCGAGTCCCACCTCG). We found that the SEV motif is contained in 34 full-length and four truncated elements, with a widespread chromosomal distribution (Figure 1 and Supplementary Table S1). Elements with SEV motif(s) are characterised as SEV-VL30s thereafter. Previously, we have systematically annotated VL30s and have calculated the integration time for individual elements [11], since the current integrations have the highest probability to be transcriptionally active and retrotransposition-competent. Importantly, most SEV-VL30s, 25/36 full-length elements, are current integrations with intact and identical LTRs (depicted with red arrows in Figure 1). 

### 3.2. Structural and Functional Properties of SEV-VL30 RNAs

**Figure 2:**
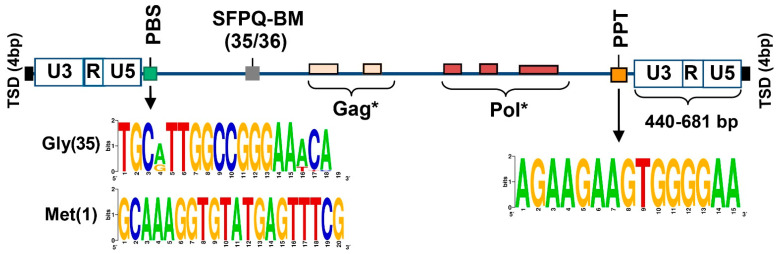
Structural properties of full-length SEV-VL30s. From left to right: TSD: Target site duplications, hallmark of integrations following Long Terminal Repeat (LTR) retrotransposition; U3-R-U5: structure of full-length LTRs (size of each LTR in base-pairs (bp) is depicted in 3′ LTR); PBS: Primer binding site, necessary for minus strand amplification during reverse transcription, including consensus sequences for the two types of tRNA species that are complementary for PBS; SFPQ-BM: Binding motifs for SFPQ protein; Gag and pol retroviral genes (* consensus sequences contain multiple stop codons and render VL30s non-autonomous retrotransposons); PPT: polypurine tract, necessary for puls strand amplification during reverse transcription.

In a later step, we systematically annotated the structural properties of SEV-VL30s. As found in the previous section, 25/36 full-length elements contain identical 5′ and 3′ LTRs, consensus sequences that are prerequisites for retrotransposition. LTRs, primer binding sites (PBS) and the polypurine tract (PPT) are the required sequence hallmarks for retrotransposition competence. Importantly, we found that all full-length elements contain both PBS and PPT sequences (Figure 2 and Supplementary Table S1). A total of 35/36 elements contain a PBS sequence that is complementary to Gly tRNAs and one element to Met tRNA. PPT is highly conserved and no mutations from the consensus sequence were detected in SEV-VL30s. A characteristic sequence of 4 bp target genomic site duplication (TSD) following retrotransposition is detected in each case, revealing recent retrotranspostition activity. The collective results show that SEV-VL30 elements contain intact sequences that enable reverse transcription and integration; namely, 5′ and 3′ LTRs, PBS and PPT. 

SFPQ binding of VL30 RNA is mediated by two distinct sequence motifs that are hallmarks of VL30 family members [11]. Importantly, we found that the vast majority of full-length SEV-VL30s (35/36) contain at least one SFPQ binding motif (Figure 2 and Supplementary Table S1). 

The results of structural and functional analysis reveal that: (1) SEV-VL30s are potent for retrotransposition, since they contain the hallmark sequences of LTR, PBS and PPT, and (2) SFPQ binding, since they contain at least one SFPQ binding motif. Collectively, the horizontal transfer of SEV-VL30s on target cells may lead to genetic and/or epigenetic disequilibrium that is associated with oncogenesis.

### 3.3. Insights into the Evolution of SEV-VL30s 

**Figure 3:**
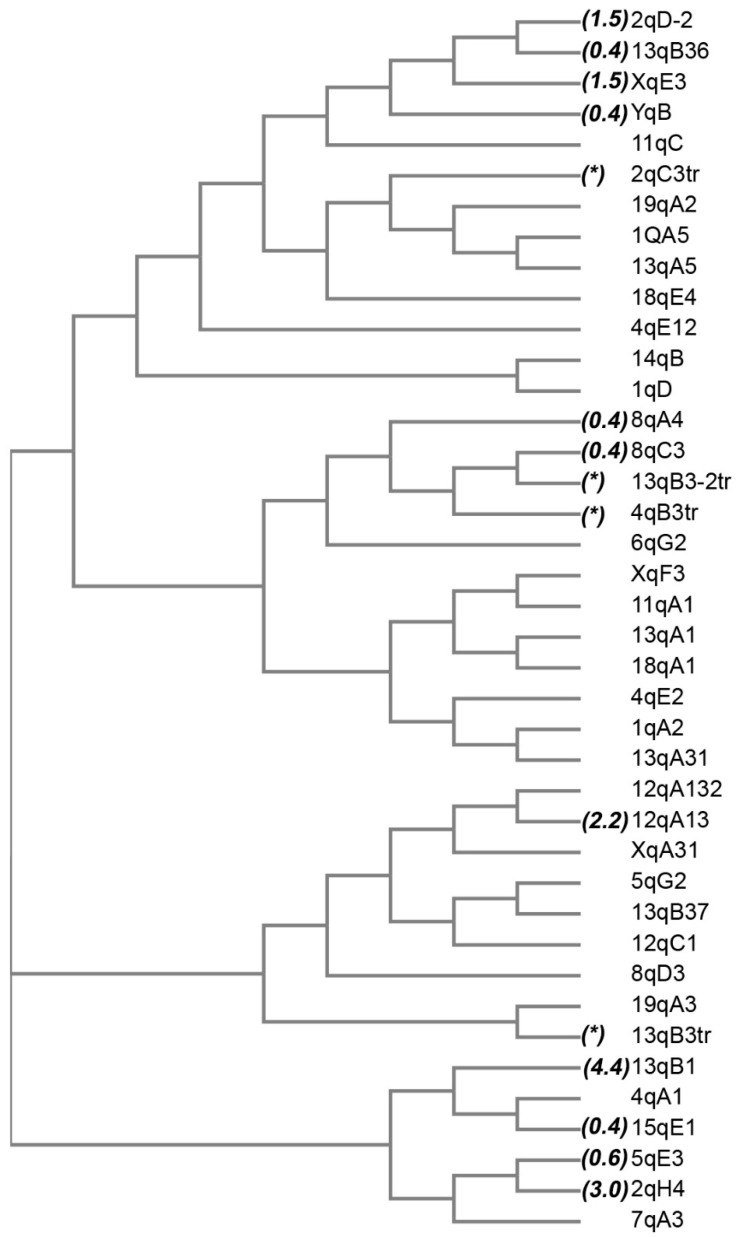
Evolutionary relationships of SEV-VL30s. The evolutionary history was inferred using the Neighbour-Joining method. The optimal tree is shown. The evolutionary distances were computed using the Maximum Composite Likelihood method and are in the units of the number of base substitutions per site. This analysis involved 40 nucleotide sequences. All ambiguous positions were removed for each sequence pair (pairwise deletion option). There were a total of 1130 positions in the final dataset. Numbers in parentheses denote integration time, while asterisk denotes truncated elements in which integration time cannot be calculated.

In order to identify the evolutionary history of SEV binding in VL30s within the mouse germline, we performed phylogenetic analysis for SEV-VL30s. Using the MUSCLE algorithm, 5′ LTRs of SEV-VL30s were aligned, and a phylogenetic tree was drawn using the Neighbour-Joining method (Figure 3). The time of integration, as has been calculated in a previous study from our group [11], is shown beside the name of each SEV-VL30 (excluding contemporary elements that are calculated as today’s integrations). The final evolutionary tree includes the combined information of VL30 integration time and the divergence of individual SEV-VL30s. The SEV motif seems to have occurred ~4.4 million years ago (MYA), and 13 qB1 VL30 is the first known to have acquired this sequence and can be characterised as the founding SEV-VL30 element. The tree depicts a first wave of expansion including 13 qB1, indicated in the lowest node of the tree, and at least four additional contemporary expansions/integrations of SEV-VL30s that lead to the spread of this feature that we observe today in the mouse genome. 

The *Mus* genus contains a number of highly similar species that diverged from a common ancestor about 10 MYA. To gain further insights into the origins of SEV enrichment features, we explored the existence of the SEV motif in the 30 known species that belong to the *Mus* genus and found that the conserved 26-nt consensus SEV motif is present only in the genomes of *Mus musculus* and *Mus spretus* (Figure S1). Importantly, the divergence time between *Mus musculus* and *Mus spretus* is ~3 MYA, which is in accordance with the result from our previous analysis that indicates an occurrence time of ~4.4 MYA. Collectively, our data indicate that SEV motifs occurred in the Mus germline ~4.4 MYA and have since expanded only in *Mus musculus* through SEV-VL30 retrotransposition.

### 3.4. Epigenetic Regulation of SEV-VL30s

In the next step, we analysed the transcriptional potential of SEV-VL30s. The database of candidate cis-regulatory elements (cCREs) integrates high-throughput epigenetic data on DNAseI digestion sites, the histone modifications of H3K4me3 and H3K27ac and CTCF ChIP-seq data produced by the ENCODE and Roadmap Epigenomics Consortia. The existence of cCREs is considered a strong indication for transcriptional activity [31]. We screened the annotated SEV-VL30s for the existence of cCREs and found that 25/40 elements reside adjacent to cCREs and evaluated their function (Table 1). The cCRE elements related to SEV-VL30s include 11 promoters, 39 proximal enhancers, 88 distal enhancers, 3 DNAse I/H3 K4me3 regions and 7 CTCF binding sites. 

**Figure 4:**
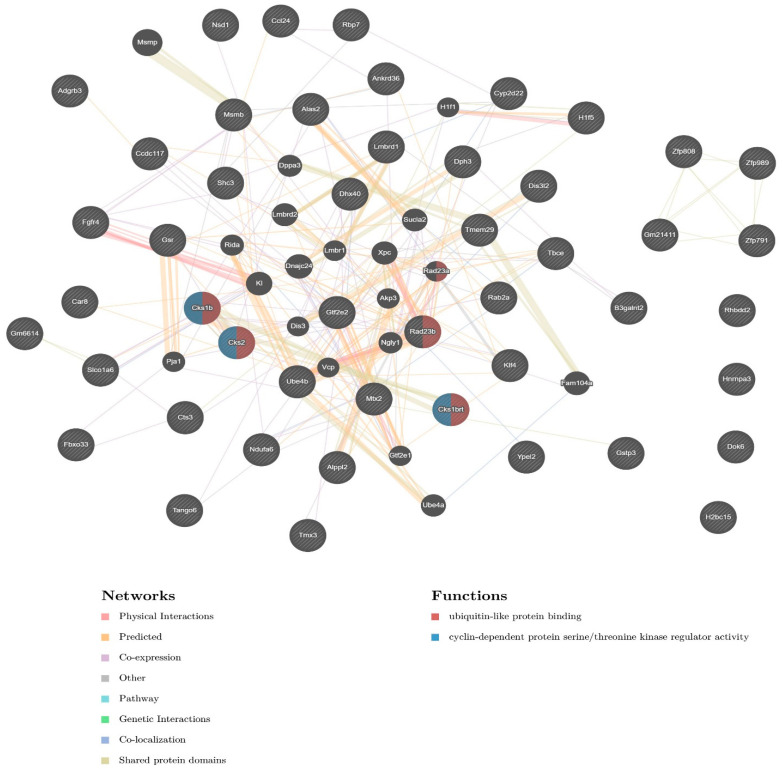
Network analysis of CRE-VL30-related genes. The final network includes 47 input genes (indicated with stripes) and 20 related genes. In total, 67 genes and 353 total links are presented. Interactions in the network are depicted in different line colours while functions are depicted in coloured circles representing individual proteins (see legend above for details).

Using the GREAT tool, which uses a combined statistical analysis to associated gene regulatory domains with genomic regions, we calculated the distance between cCRE-related SEV-VL30s and transcription start sites (TSSs) of the nearest mouse genes. The 25 elements reside in the vicinity of TSSs from 47 mouse genes, in distances as near as 4429 bp (Figure S2). To further evaluate the regulatory impact of potentially active CRE-VL30s, we generated a regulatory network containing the 47 CRE-VL30-related genes (Figure 4). Importantly, the majority of the genes in the inferred network physically interact and a percentage of them are associated with ubiquitin–protein binding (Rab23a, Rab23b, Csk1b, Csk2) as well as cyclin-dependent protein phosphorylation (Csk1b, Csk2, Csk1brt). GREAT analysis of non-SEV-associated VL30s revealed a different dataset of 510 genes. Only Gtf2e2 and Gsr were common among the two gene sets (Figure S3). 

To further evaluate possible shared functions of SEV-VL30 genes, we performed enrichment analysis in Enrichr. Based on data from MGI Mammalian Phenotype Level 4 2021, we found that our dataset is enriched, among others, in the following datasets (Figure S4): embryonic lethality prior to organogenesis (MP:0013292), abnormal embryo size (MP:0001697), increased cardiac cell glucose uptake (MP:0030018), increased prostate intraepithelial neoplasia incidence (MP:0009219), abnormal gastrulation movements (MP:0002174) and preweaning lethality (MP:0011100). Next, we analysed the enriched pathways for the dataset of 510 non-SEV VL30 genes (Figure S5). The enriched pathways of SEV-VL30 genes were found to be distinct to those associated with non-SEV VL30s. 

Collectively, most SEV-VL30s reside in transcriptionally active regions, as characterised by cCREs. Nearby genes are transcribed into proteins that physically interact and their expression is associated with embryonic abnormalities and neoplasia. The genomic distribution of SEV-VL30s is distinct to the remaining non-SEV elements. We conclude that SEV-VL30s are related to specific cellular functions, distinct from non-SEV VL30s.

## 4. Discussion

In this study, we established the origins of VL30s that are packaged in SEVs and proved that they formed a distinct functional group of 40 elements within the VL30 family. SEV enrichment evolved ~4.4 MYA and expanded as an inherent hallmark of the VL30 family along with recent VL30 expansions through retrotransposition. Most current SEV-VL30 integrations contain hallmarks of active transcription, retrotransposition competency and are associated with developmental abnormalities and neoplasia. In conclusion, the potential of VL30 lncRNAs through SEVs suggests a significant pathophysiological role in target cells. 

The SEV-VL30 lncRNAs are associated with cancer with at least two mechanisms. First, high-frequency retrotransposition as a result of stress is associated with epithelial-to-mesenchymal transition and cancer stem cell formation [19]. Second, SFPQ binding is associated with oncogene transcription and cancer induction [43]. In our study, we confirm that all SEV-VL30s contain the sequence hallmarks for retrotransposition induction, while 35 SEV-VL30s also contain SFPQ binding motifs. Thus, SEV-VL30s can be transcribed as oncogenic lncRNAs that also encode the potential to spread through SEV formation. The horizontal transfer of VL30 RNAs on target cells that may endocytose such SEVs may lead to oncogenesis through retrotransposition and/or SFPQ binding. Baris et al. suggest that SEV formation is an effective mechanism for the clearance of potentially toxic RNAs [23]. We cordially agree with this notion. The potent interferon I response against VL30-SEVs is also consistent with an antiviral immune stimulation, which may pose a defensive mechanism against the horizontal transfer of oncogenic VL30 RNAs through SEVs. Further studies would warrant the impact of the interplay between SEVs containing VL30 RNAs and host cell mechanisms. 

VL30 elements appeared in the *Mus* germline ~17 MYA and the largest wave of expansion is calculated at <1 MYA, resulting in 86 full-length elements in today’s mouse genome [11]. We suggest that the SEV motif was “embedded” in a VL30 integration, most probably 13qB1, before the main wave of VL30 expansions through retrotransposition, which ultimately led to the establishment of SEV enrichment as a hallmark of the VL30 family. In a previous report, we have demonstrated that SFPQ binding is a universal feature of VL30 elements and has spread in parallel to the VL30 expansions >17 million years ago [11]. Therefore, most VL30s contain SFPQ binding sites, while less contain an SEV enrichment motif, calculated in the current report to have occurred 4.5 MYA. Ultimately, both structural features have been expanded through retrotransposition events, connecting epigenetic regulation through SFPQ binding to horizontal gene transfer through extracellular vesicles.

The transcriptional profile of individual VL30s is known to be cell-type-specific [44] and inducible upon several types of stress, including oncogenic and oxidative stress as well as hormonal stimulation [16,17,18]. In the current study, we assessed the transcription competency of SEV-VL30s and found that the majority are associated with candidate cis-regulatory elements (cCREs) by ENCODE [31]. Twenty-five SEV-VL30s adjacent to cCREs reside in the vicinity of 47 mouse genes. Importantly, most of the SEV-VL30s reside less than 50Kb away from the respective transcription start sites (Figure S2), a distance that is in accordance with the concept that 80% of known promoter and enhancer interactions occur in a window of <320 Kb, as calculated by the analysis of Hi-C data [45]. Based on this notion, SEV-VL30s that reside in cCREs have the potential to influence the expression of their nearby target genes. Following GREAT and Enrichr analysis of non-SEV VL30s, we found that SEV-VL30s are related to specific cellular functions, distinct from non-SEVs. Network analysis revealed that most SEV-VL30-related genes interact, and pathway enrichment analysis showed that they are associated with developmental abnormalities and neoplasia. 

Our results agree with the conceptual framework that retrotransposon induction is associated with the orchestration of regulatory networks, such as the ones observed during early human development that assist stem cell pluripotency [46]. The balance between the induction of abnormalities and the establishment of novel regulatory networks is also consistent with this notion, suggesting an interaction with the host that includes cycles of restraint and rehabilitation [47]. The potential of targeting the genome of the cell in which VL30 lncRNA is derived as well as the genomes of other cells, through efficient enrichment in SEVs, represents a novel paradigm of interaction with the host, with many exciting implications for non-coding RNA biology.

## Figures and Tables

**Table 1:** CRE-VL30s adjacent to cCREs.

CRE-VL30	Nearest Genes (Distance to TSS)	cCRE Type/Function
**1qA5**	Lmbrd1 (+338,654), Adgrb3 (+812,423)	enhD (2X)
**1qD**	Alppl2 (+11,819), Dis3l2 (+374,273)	enhD, CTCF
**2qC3tr**	Hnrnpa3 (−18,737), Mtx2 (+814,721)	enhP (4X), enhD (5X)
**4qA1**	Car8 (−156,331), Rab2a (−140,272)	enhD
**4qB3tr**	Rad23b (+70,666), Klf4 (+111,757)	CTCF
**4qE1-2**	Zfp989 (−32,805), Gm21,411 (−29,581)	K4m3, enhP
**4qE2**	Ube4b (−9840), Rbp7 (+18,389)	Prom, enhP (5X), enhD (6X)
**5qG2**	Ccl24 (−53,256), Rhbdd2 (−6313)	Prom, enhP (2X), CTCF (2X), K4m3
**6qG2**	Gm6614 (−158,676), Slco1a6 (+16,063)	enhP, enhD
**8qA4**	Gtf2e2 (−16,097), Gsr (+63,004)	enhD (3X)
**8qC3**	Zfp791 (−16,620), Cks1brt (−12,209)	enhD (2X)
**8qD3**	Tango6 (−4073)	prom, EnhP, enhD (2X), CTCF (2X)
**11qA1**	Alas2 (−59,876), Tmem29 (−28,349)	enhP (4X), enhD (3X)
**11qC**	Ankrd36 (−14,253), Ccdc117 (−13,244)	prom, enhP (2), enhD (4X)
**12qC1**	Dhx40 (−6323), Ypel2 (+179,638)	Prom, enhP (5X), enhD (2X)
**13qA1**	Fbxo33 (−5156)	enhD (6X)
**13qA3-1**	Tbce (+16,998), B3galnt2 (+68,171)	prom (3X), enhP (2X), enhD (2X)
**13qA5**	Hist1h2bn (+11,370), Hist1h1b (+15,132)	prom, enhP (5X), enhD, K4m3
**13qB1**	Shc3 (−69,417), Cks2 (−8731)	enhD (7X), CTCF
**13qB3tr**	Nsd1 (−22,938), Fgfr4 (+34,204)	enhD (3X)
**14qB**	Cts3 (−517,722), Zfp808 (−42,067)	enhD (7X)
**15qE1**	Dph3 (−32,316), Msmb (−24,101)	prom (2X), enhP (4X), enhD (7X)
**18qE4**	Ndufa6 (−10,978), Cyp2d22 (+14,991)	enhD (3X)
**19qA2**	Tmx3 (−672,104), Dok6 (−68,522)	prom (2X), enhP (3X), enhD (16X)
**XqF3**	Gstp3 (−4429)	enhD (3X)

* cCRE type: **Prom**: Promoter; **enhP**: proximal enhancer; **enhD**: distal enhancer; **CTCF**: CTCF binding site; **K4m3**: DNAse I/H3 K4me3 region.

## Supplementary Materials

The following are available online at https://www.mdpi.com/article/10.3390/biomedicines9111742/s1, Figure S1: SEV-VL30s in the phylogenetic tree of the Mus genus. Figure S2: Distance of SEV-VL30s to mouse genes promoters. Figure S3: Distance of VL30 elements not associated with SEVs (non-SEV VL30s) to mouse genes promoters, following GREAT analysis. Figure S4: Enrichment for mammalian phenotypes in genes related to SEV-VL30s. Figure S5: Enrichment for mammalian phenotypes in genes related to to non-SEV VL30s. Table S1: Structural features of SEV-VL30s.
