# Supplementary material for: Origins and Function of VL30 lncRNA Packaging in Small Extracellular Vesicles: Implications for Cellular Physiology and Pathology
Source: Biomedicines. 2021 Nov 22;9(11):1742. doi: 10.3390/biomedicines9111742 (PMC8615591; doi:10.3390/biomedicines9111742)
Supplement: Supplementary file 1 [file biomedicines-09-01742-s001.zip › Suplementary Figures.pdf]

Suplementary Figures

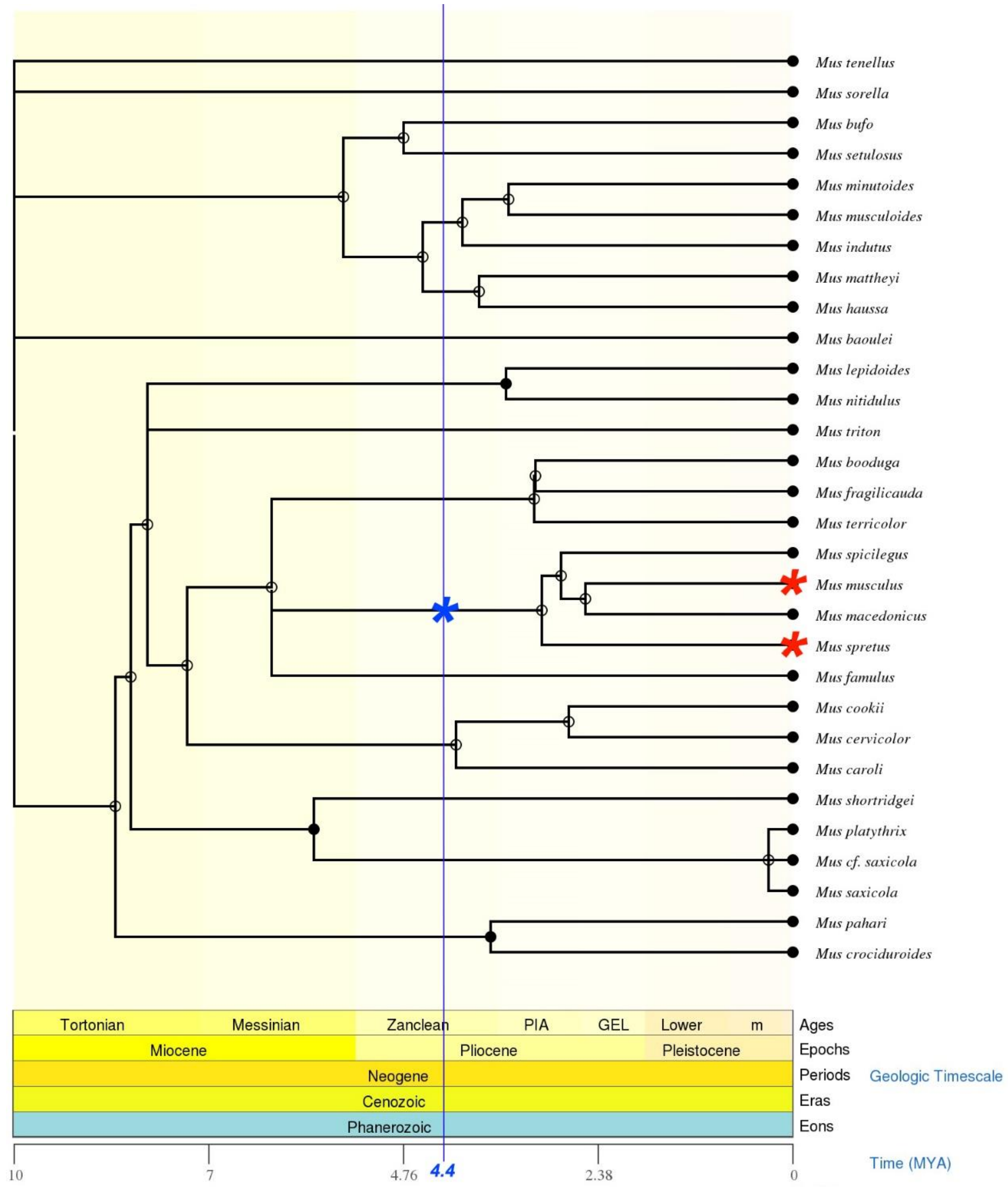

Figure S1: SEV-VL30s in the phylogenetic tree of the *Mus* genus. The two *mus* species containing SEV-motifs are indicated with red asterisk. Blue asterisk denotes the inferred evolutionary time of SEV-motif occurrence of 4.4 million years ago (MYA), which is also indicated in the timeline with blue vertical line.

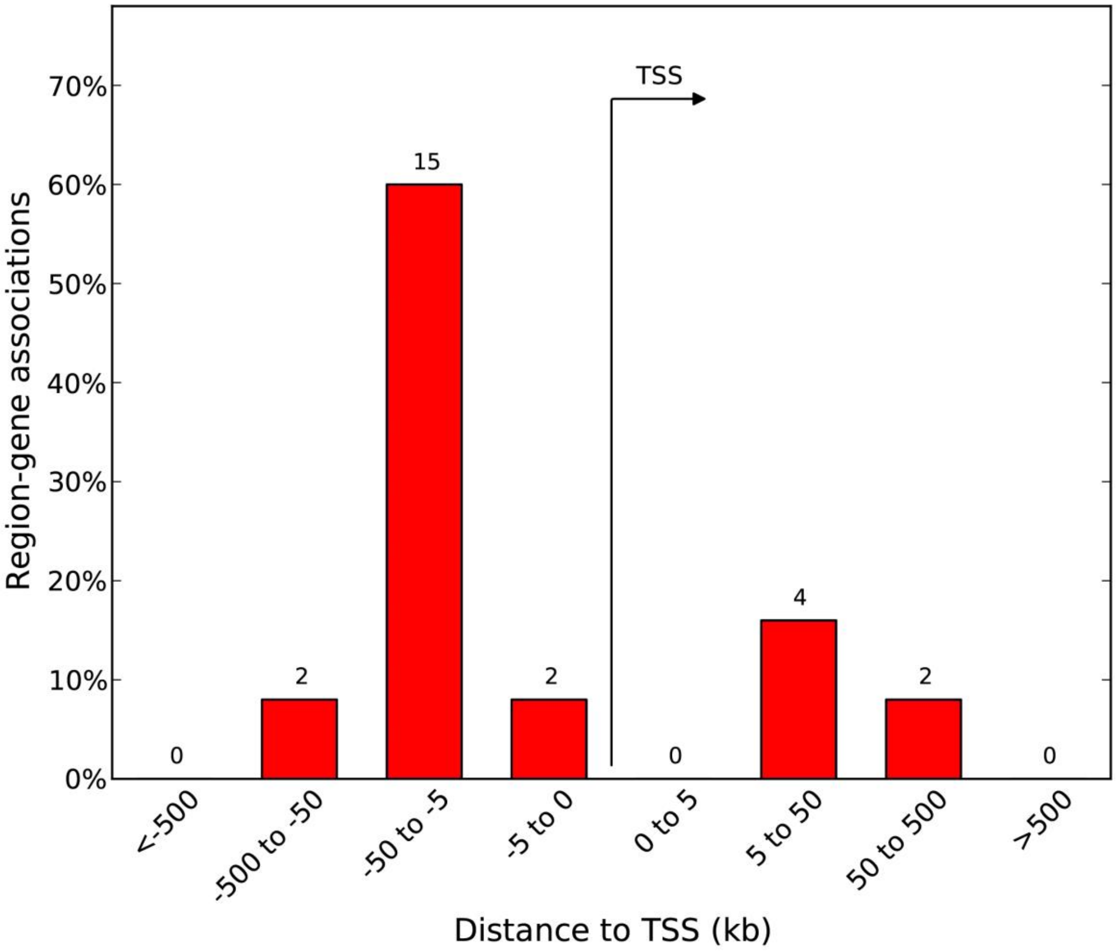

Figure S2: Distance of SEV-VL30s to mouse genes promoters. The distance between SEV-VL30s, associated to cCREs and mouse Transcription Start Sites (TSS) was calculated following GREAT analysis. The percentage and number of SEV-VL30s in different distances to Transcription Start Sites (TSS) is shown. Relative distance to TSS is divided into 0-5, 5-50, 50-500 and >500 kb.

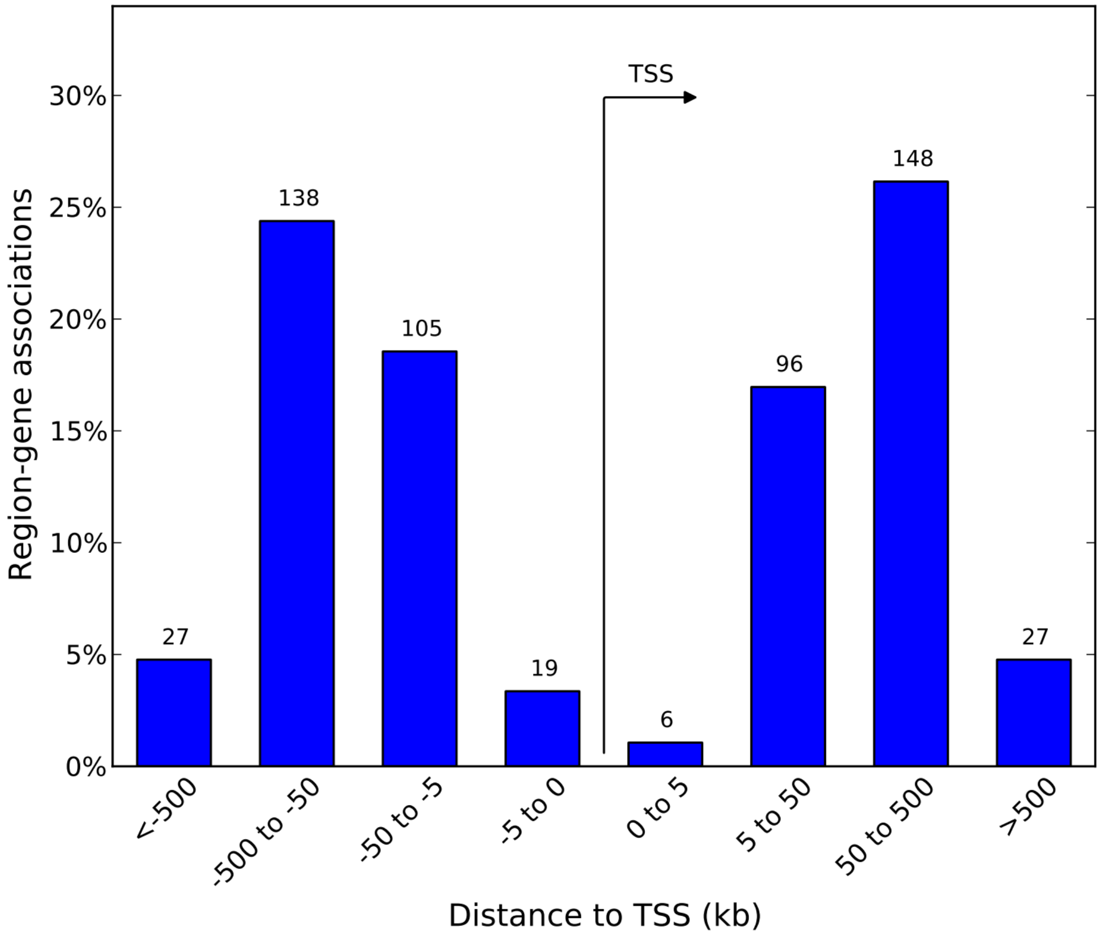

Figure S3: Distance of VL30 elements not associated with SEVs (non-SEV VL30s) to mouse genes promoters, following GREAT analysis. The percentage and number of SEV-VL30s in different distances to Transcription Start Sites (TSS) is shown. Relative distance to TSS is divided into 0-5, 5-50, 50-500 and >500 kb.

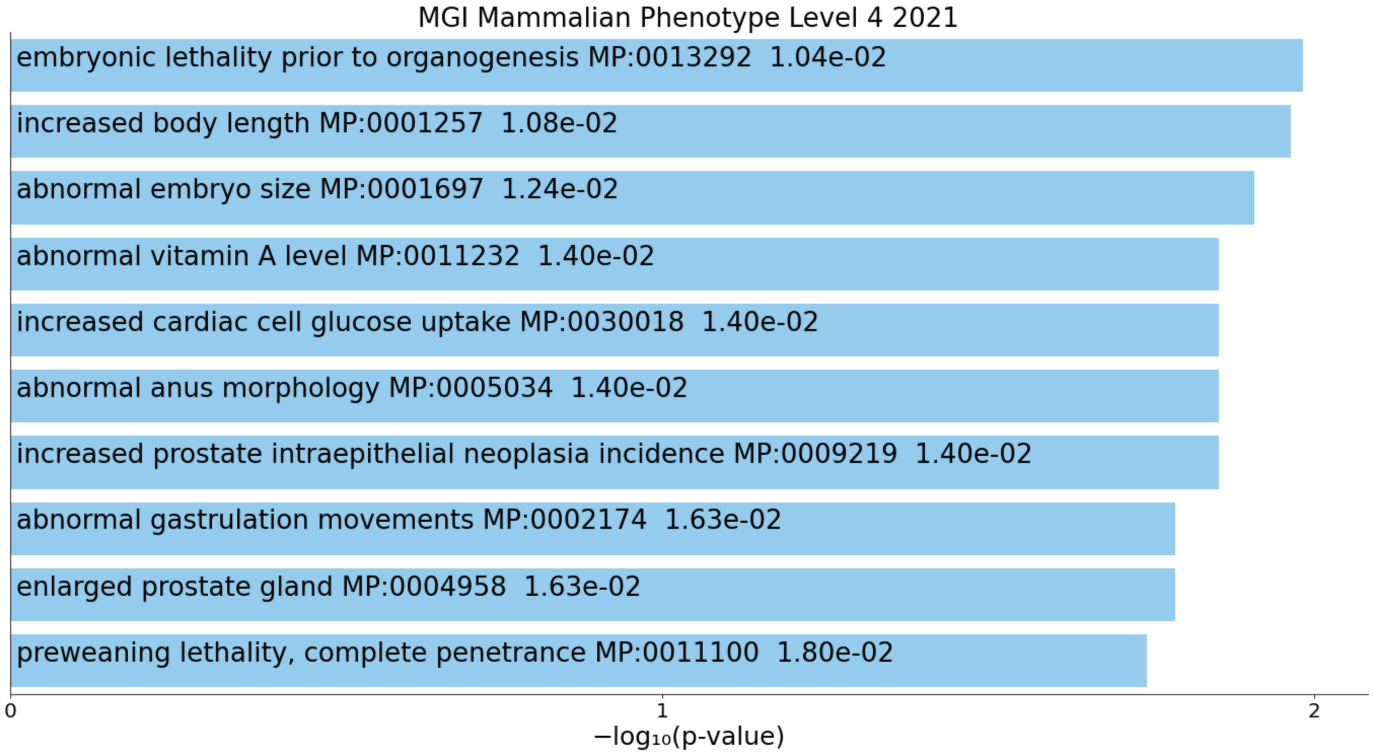

Figure S4: Enrichment for mammalian phenotypes in genes related to SEV-VL30s. Following Enrichr analysis, the top-10 phenotypes containing SEV-VL30s related genes were plotted, based on significance p-value.

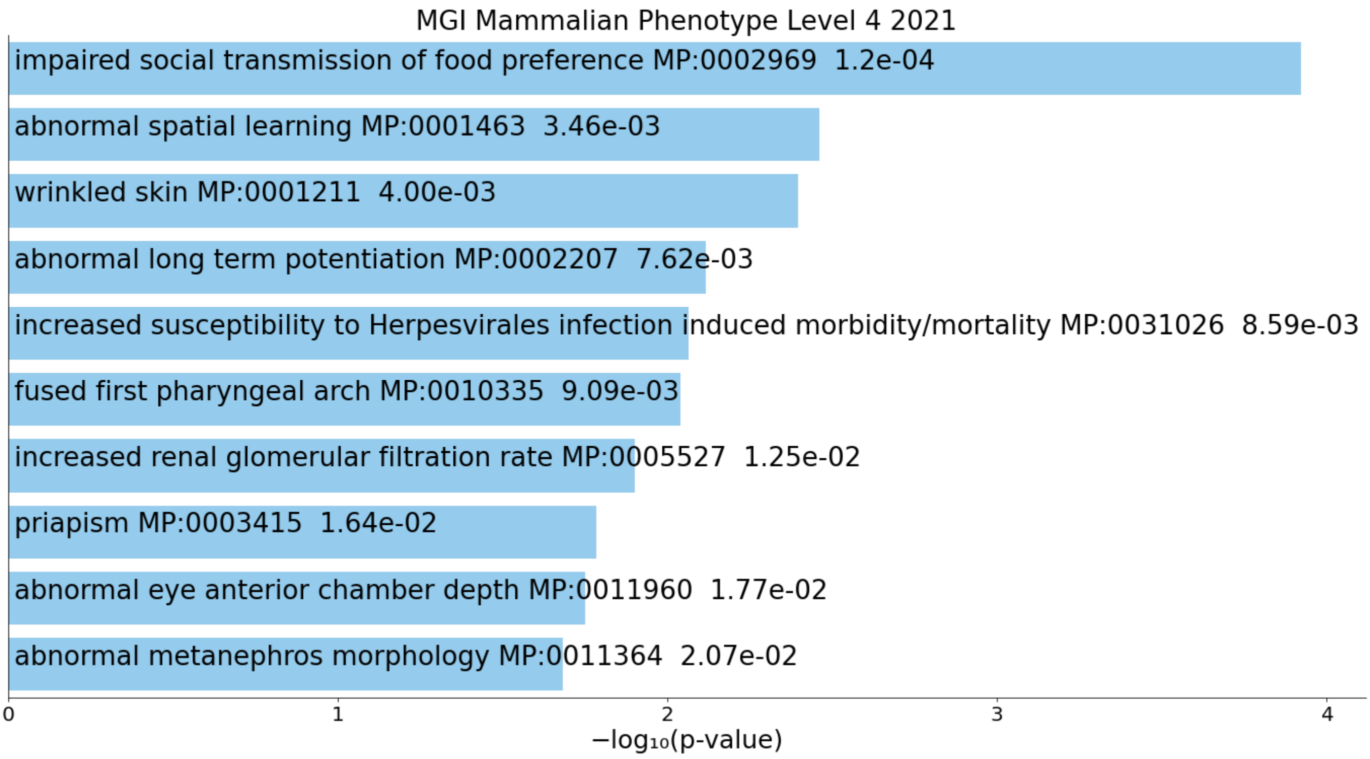

Figure S5: Enrichment for mammalian phenotypes in genes related to to non-SEV VL30s. Following Enrichr analysis, the top-10 phenotypes containing non-SEV VL30s related genes were plotted, based on significance p-value.
